# Supplementary material for: GWGGI: software for genome-wide gene-gene interaction analysis
Source: BMC Genet. 2014 Oct 16;15:101. doi: 10.1186/s12863-014-0101-z (PMC4201693; doi:10.1186/s12863-014-0101-z)
Supplement: Additional file 1: Table S1. — The summary of the top SNPs selected by GWGGI. [file 12863_2014_101_MOESM1_ESM.pdf]

Table S1: The summary of the top SNPs selected by GWGGI

	T1D		CAD	
	LRMW	TAMW	LRMW	TAMW
SNPs	rs3957146	rs9273363*	rs2416472	rs4970605
	rs377763*	rs3135377*	rs7628245	rs890447
	rs9270986[#]	rs9270986[#]	rs7723508	rs7628245
	rs9273363*	rs3957146	rs159171	rs2416472
	rs3177928[#]	rs3129900[#]	rs890447	rs159171
	rs2647046[#]	rs3916765*	rs9327048	rs4819660
	rs6679677**	rs2894249*		rs16883114
		rs3129932*		rs6475606**
		rs9275418*		rs11640295
		rs910049**		rs6531531
		rs3129768[#]		rs1333049**
		rs3132959*		rs1333048*

**The SNPs have been previously reported to be associated with T1D or CAD.

*The SNPs have been previously reported to be associated with traits related to T1D or CAD in comorbidity analyses, pathway analyses or interaction analyses.

[#]The SNPs have been previously reported to be associated with other complex diseases.
